# Soluble cytokines enhance risk prediction across all stages of classical Hodgkin lymphoma

**DOI:** 10.1186/s40364-025-00881-0

**Published:** 2026-01-15

**Authors:** Alexandra Kredátusová, Tomáš Chupáň, Heidi Móciková, Alice Sýkorová, Jana Marková, Marie Lukášová, Ľubica Gahérová, Pavla Štěpánková, Eva Kriegová, Mária Maco, Tomáš Kozák, Tomáš Papajík, Stephen M. Ansell, Vít Procházka

**Affiliations:** 1 Department of Hemato-Oncology, Faculty of Medicine and Dentistry, Palacky University and University Hospital Olomouc, Olomouc, Czech Republic; 2 Innovation and Digitalisation Department, University Hospital Olomouc, Olomouc, Czech Republic; 3 Department of Hematology, University Hospital Kralovske Vinohrady and 3rd, Faculty of Medicine, Charles University, Prague, Czech Republic; 4 4th Department of Internal Medicine - Haematology, Faculty of Medicine, University Hospital Hradec Králové, Hradec Kralove, Czech Republic; 5 Department of Immunology, Faculty of Medicine and Dentistry, Palacky University and University Hospital Olomouc, Olomouc, Czech Republic; 6 Division of Hematology, Mayo Clinic, Rochester, MN USA

## Abstract

Classical Hodgkin lymphoma (cHL) is a heterogeneous malignancy with favorable outcomes, but accurate prognostic stratification remains challenging, particularly across all disease stages. Traditional risk models are focused on advanced stages and do not account for tumor microenvironment (TME) dynamics. Soluble cytokines reflecting TME activity may offer additional prognostic value. In our prospective multicenter study, we investigated the prognostic value of pretreatment plasma levels of soluble TARC, sCD30, sCD163, and sIL-6 in 162 newly diagnosed cHL patients and developed a model incorporating these biomarkers for risk prediction across all stages. Cytokine levels were measured using ELISA, and clinical characteristics, treatment responses, and outcomes were collected. The primary endpoint was 5-year progression-free survival (PFS). Elevated levels of sIL-6 and sCD30 were associated with higher disease stage, presence of B-symptoms, extranodal involvement, and inferior 5-year PFS. A novel prognostic model incorporating age, extranodal disease, and high sCD30/sIL-6 levels outperformed IPS-3 in predicting therapy failure. Patients with both elevated sCD30 and sIL-6 levels at diagnosis had significantly worse outcomes. Integrating soluble cytokine biomarkers, particularly sIL-6 and sCD30, into prognostic models enhances risk stratification across all stages of cHL and supports future efforts toward biomarker-driven, personalized treatment strategies.

**Supplementary Information:**

The online version contains supplementary material available at 10.1186/s40364-025-00881-0.

## To the editor,

**Fig. 1:**
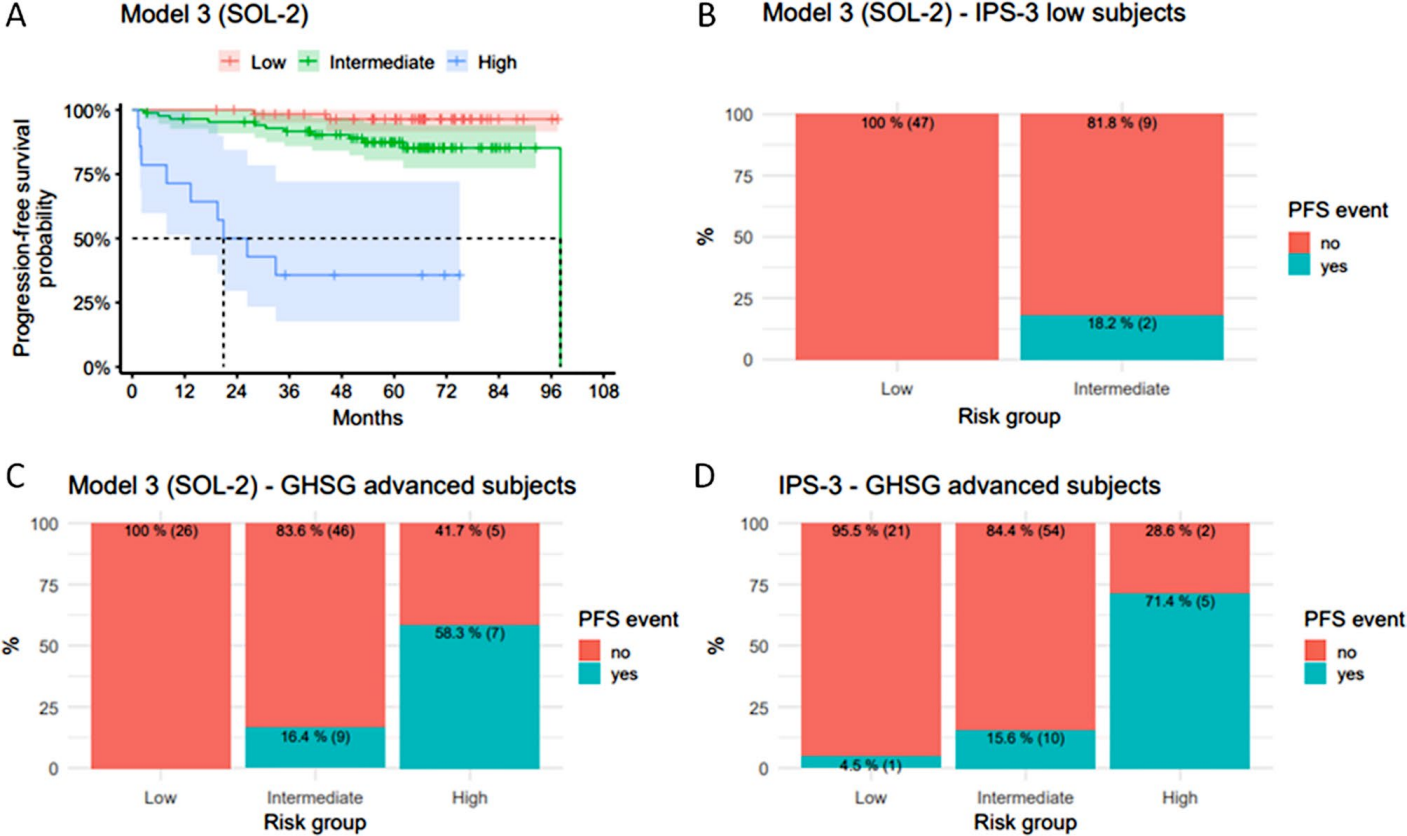
(**A**) Progression-free survival (all patients) stratified according to the Model 3. (**B**) The distribution of PFS events across IPS-3 low risk pts according to the Model-3 risk groups. (**C**) Progression-free survival (GSHG-advanced pts) stratified according to the Model 3. (**D**) Progression-free survival (GSHG-advanced pts) stratified according to the IPS-3

we conducted a prospective multicenter study to evaluate soluble cytokines as predictors of outcomes in classic Hodgkin lymphoma (cHL). cHL has a generally favorable prognosis, however, some patients, even in the intermediate stage, experiences disease progression or relapse. This highlights the need for a prognostic model applicable across all disease stages to refine risk stratification and guide treatment decisions. We aimed to integrate clinical features with soluble cytokine markers to create a more precise prognostic tool. We hypothesized that soluble biomarkers in plasma reflect the tumor microenvironment (TME), and while linked to immune evasion and tumor growth in cHL [1–3], influence tumor behavior and patient outcomes. The goal of our study was to incorporate these markers into a model capable of predicting progression-free survival (PFS) in cHL patients, irrespective of clinical stage. A total of 162 patients diagnosed with cHL, with a median age of 42 years, were recruited from three institutions within Czech Hodgkin Lymphoma Study Group. The cohort included patients with various stages based on the German Hodgkin Study Group (GHSG) classification: 16% of patients were categorized as having early-stage disease, 25% as intermediate, and 59% as advanced. During a median follow-up period of 64.1 months (1.3–111.0), twenty-three PFS-related and thirteen OS-related events were observed. The five-year PFS rate was 86% (95% CI: 0.81–0.92), and 92% of patients (95% CI: 0.88–0.96) remained alive at five years without an OS event. The primary aim was to develop a complex prognostic model to predict PFS and stratify patients into low, intermediate, and high-risk categories. We identified age, albumin levels, and extranodal involvement as key variables for **Model 1**, which was further refined by incorporating soluble cytokines (sIL-6, sCD30, sCD163, and TARC) [4]. Elevated levels of sCD163 and sIL-6 or sCD30 and sIL-6 were linked to significantly worse PFS. We also explored alternative models incorporating cytokine combinations, resulting in **Models 2** and **3**. These models retained age and extranodal involvement, replacing albumin levels with cytokine pairs. Models 2 and 3 showed comparable Akaike Information Criterion (AIC) and C-index values to **Model 1**. Cross-validation confirmed that **Model 3**, which included the sCD30/sIL-6 high-high cytokine combination, outperformed the IPS-3 score [5] in predicting patient outcomes, particularly for advanced-stage patients (Table 1). For clinical applicability, we discretized the linear predictors from **Models 1–3** into three risk groups using the rsolr12() function. **Model 3**, applying the SOL-2 algorithm, showed the best separation in Kaplan–Meier survival curves (Fig. 1A). Notably, **Model 3** also provided finer stratification than IPS-3, particularly for low-risk patients classified by IPS-3, suggesting that our model may identify high-risk patients who would benefit from more intensive treatment despite their low IPS-3 score (Fig. 1B). Further validation against IPS-3 in a subgroup of GHSG advanced-stage patients demonstrated that **Model 3** could better differentiate between intermediate- and high-risk patients compared to IPS-3 (Fig. 1C-D). To facilitate clinical application, an online calculator was developed to simplify the use of our model [6]. Our study has some limitations, including the relatively low number of high-risk patients due to the generally good treatment outcomes in cHL. A larger cohort is needed for further validation. The selection of cytokines is another limitation; other cytokines may also contribute to disease aggressiveness, which could be explored in future studies. TARC, although correlated with tumor burden, did not emerge as significant in the model, possibly reflecting disease activity rather than tumor aggression. This suggests that TARC may be more useful for monitoring disease activity than predicting patient outcomes [7, 8]. The strength of this study lies in its prospective design and multicenter approach, which enhances generalizability. All laboratory measurements were performed centrally using standardized ELISA methods, ensuring data consistency. Future studies should validate our model in larger cohorts treated with novel therapies such as brentuximab vedotin or immune checkpoint inhibitors. Combining soluble biomarkers with genetic profiling, such as cell-free DNA analysis, could offer a more comprehensive prognostic tool, enhancing personalized treatment strategies. In conclusion, soluble biomarkers like sIL-6 and sCD30, integrated into prognostic models, can improve risk stratification in cHL, particularly for borderline-stage disease or patients with unrecognized high-risk features. Our findings support the development of more individualized, risk-adapted strategies beyond traditional staging systems.

**Table 1:** Comparison of the models’ performance

Model	IBS (data)	IBS (CV)	C-index (data)	C-index (CV)
Reference	0.093	0.093	–	–
Model 1	0.067	0.074	0.83	0.79
Model 2	0.069	0.076	0.81	0.78
Model 3	0.069	0.076	0.80	0.76
Model 4 = IPS-3 score only	0.073	0.077	0.72	0.70

Note: IBS = Integrated Brier Score, a measure of prediction error for survival models. The C-index measures how well the model discriminates between patients. It represents the probability that, for any two patients, the one who experiences the event earlier had a higher predicted risk according to the model. A C-index of 0.5 indicates no predictive ability (equivalent to chance), whereas a C-index of 1.0 reflects perfect discrimination. The AIC quantifies model quality by balancing fit and complexity (lower AIC indicates a better-fitting, more parsimonious model)

## Supplementary Information

Below is the link to the electronic supplementary material.

Supplementary Material 1

## Data Availability

The dataset analysed during the current study is available in the Zenodo repository, 10.5281/zenodo.16792459.

## Abbreviations

AIC: Akaike Information Criterion
cHL: classical Hodgkin lymphoma
GHSG: German Hodgkin study group
IPS: 3–Simplified International Prognostic Score
OS: Overall survival
PFS: Progression–free survival
TME: Tumor microenvironment
