# Supplementary material for: Soluble cytokines enhance risk prediction across all stages of classical Hodgkin lymphoma
Source: Biomark Res. 2026 Jan 15;14:14. doi: 10.1186/s40364-025-00881-0 (PMC12809970; doi:10.1186/s40364-025-00881-0)
Supplement: Supplementary file 1 — Supplementary Material 1 [file 40364_2025_881_MOESM1_ESM.docx]

**Patients, methods and statistical analysis**

*1 Study design*

This prospective, multicentric non-interventional observational study was conducted on behalf of the Czech Hodgkin Lymphoma Study Group (CHSG; NCT06263530, University Hospital Královské Vinohrady). We included 162 unselected patients aged 18 years or older with newly diagnosed cHL treated at the hemato-oncology departments of three university hospitals in the Czech Republic between 1/2014 and 2/2025. Clinical, laboratory, and outcome data were collected from the prospective CHSG registry.

*2 Cytokine analyses*

Plasma samples were obtained before the start of any treatment. All samples were analyzed centrally (Olomouc) using ELISA for lymphoma cells-related biomarkers (TARC pg/ml; sCD30, ng/ml), macrophages-related biomarkers (sCD163, ng/ml), and inflammation-related cytokines (sIL-6, pg/ml) using commercially available kits: Human CCL17/TARC Quantikine ELISA Kit (RandD), Human IL-6 Quantikine ELISA Kit (RandD), Human CD163 ELISA Kit – Quantikine (RandD), Human CD30/TNFRSF8 ELISA Kit (Novus Biologicals), according to the manufacturer's instructions.

*3 Statistical Methods*

The data were analyzed using the RStudio version 2024.12.1.563. The Kaplan-Meier method was used to calculate survival probabilities. The log-rank test was used to compare patient subgroups' survival times. The significance level was set at 0.05; 2-tailed tests were used in all calculations. The differences between groups were analyzed using the Mann-Whitney U Test/ Kruskal-Wallis test (for quantitative variables) or Fisher's exact test (for qualitative variables). Spearman's rank correlation coefficient was used to assess the strength and direction of monotonic associations between continuous variables. Progression-free survival (PFS) was defined as the time from histologic diagnosis to disease relapse, progression, or death from any cause. Overall survival (OS) was measured from histologic diagnosis to death, regardless of cause.
A stepwise Cox proportional hazards regression was used to develop a predictive model for progression-free survival, with variable selection guided by Akaike Information Criterion (AIC) minimization. The initial model included relevant continuous and binary clinical variables, and analysis was restricted to complete cases (N = 157). The final model retained variables that met statistical and proportional hazards assumptions. Additional biomarkers were evaluated separately based on biological relevance. Continuous biomarker levels were dichotomized by optimizing log-rank statistics, with cutpoint selection constrained by a minimum group size ('minprop') to ensure clinical interpretability. Sensitivity analyses were performed across a range of 'minprop' values to support robust threshold determination.

*4 Patients characteristics*

Baseline characteristics of the cohort are summarized in **Supplementary Table 1**.

**Supplementary Table 1. Clinical characteristics**

| **Total** | 162 patients |
| --- | --- |
| **Median Age** | 42(18-83) Years |
| **Sex** |  |
| Male | 78 (48%) |
| Female | 84 (52%) |
| **Subtype** |  |
| Nodular sclerosis | 83 (54%) |
| Mixed cellularity | 58 (38%) |
| Lymphocyte-rich | 8 (5.2%) |
| Lymphocyte-depleted | 1 (0.6%) |
| Unknown | 12 (7.4%) |
| **Extranodal disease present** | 54 (33%) |
| **B-symptoms present** | 94 (58%) |
| **Ann Arbor Stage** |  |
| I | 17 (10%) |
| II | 60 (37%) |
| III | 36 (22%) |
| IV | 48 (30%) |
| Unknown | 1 (0.6%) |
| **GHSG risk group** |  |
| Early | 25 (16%) |
| Intermediate | 39 (25%) |
| Advanced | 93 (59%) |
| Unknown | 5 (3.1%) |
| **First-line treatment** |  |
| ABVD | 72 (46%) |
| BEACOPPesc | 66 (62%) |
| COPP/ABV | 13 (8%) |
| Other | 11 (6.8%) |

*5 Cytokine analyses*

A detailed overview of key laboratory parameters—including leukocyte and lymphocyte counts, albumin and hemoglobin levels—as well as baseline serum cytokine concentrations (TARC, sCD30, sCD163, and sIL-6) is provided in **Supplemetary** **Fig.1**, which also includes corresponding distribution summaries and boxplots. Correlations with clinical and laboratory findings are shown in **Supplemetary** **Fig**.**2**

**Supplemetary** **Fig.1 Laboratory parameters.**

**
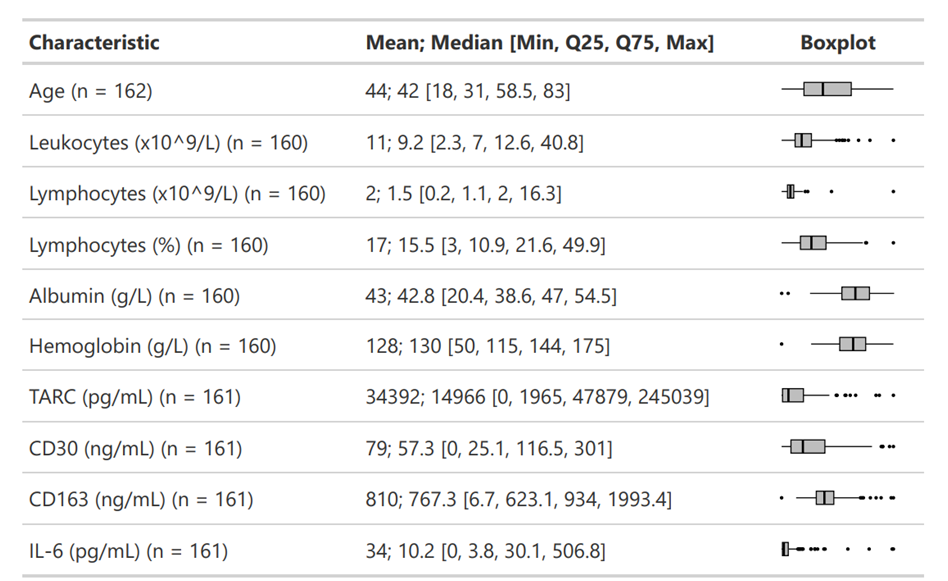
**

**Supplemetary** **Fig. 2 Correlations between clinical and quantitative laboratory findings.**

**
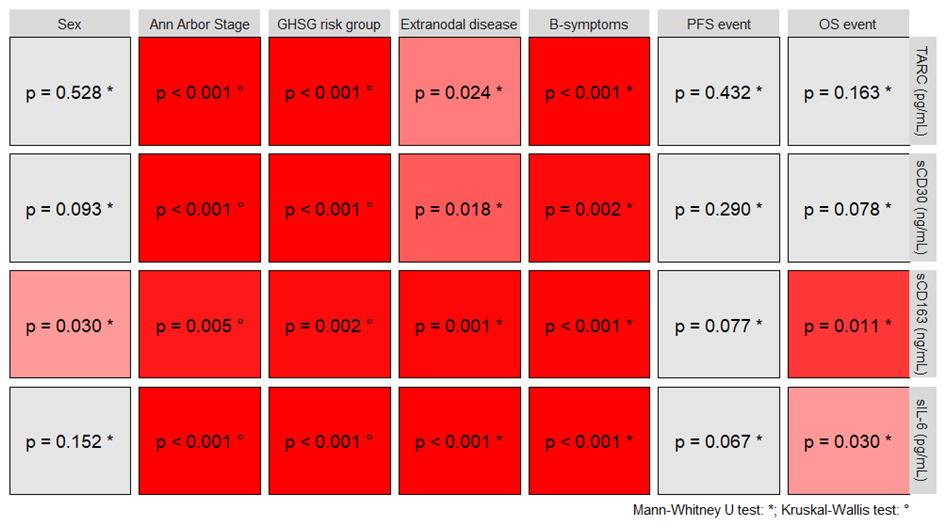
**

*6 Survival Analyses*

Kaplan–Meier survival curves illustrating these outcomes are shown in **Supplemetary Fig. 3 and Supplementary Fig. 4**, respectively.

**Supplemetary Fig.3 Overall Survival.**

**
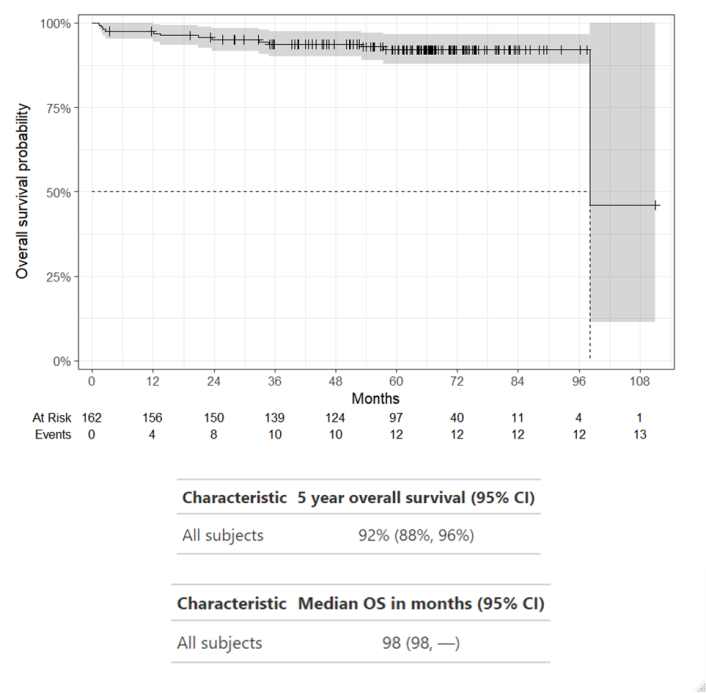
**

**Supplemetary Fig.4 Progression-free survival.**

**
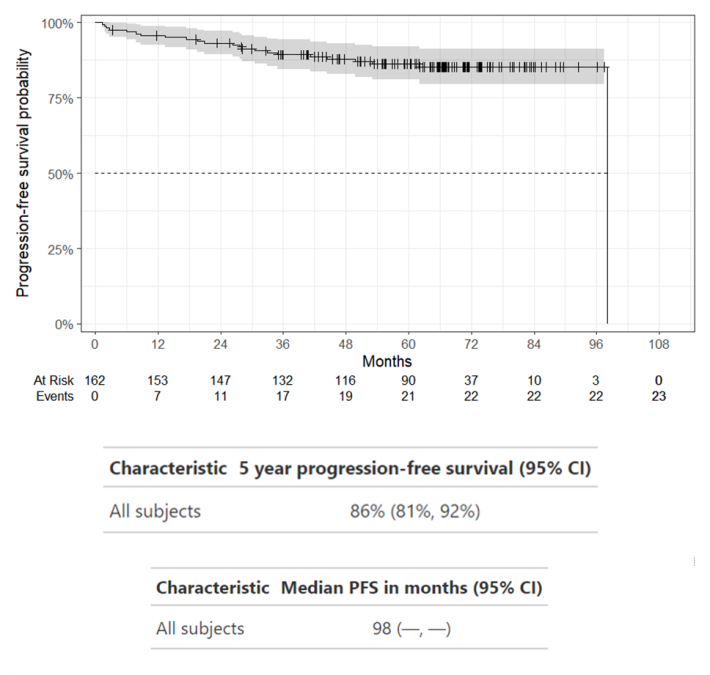
**

*7 Model development*

The primary objective was to construct a predictive model for PFS capable of stratifying patients into three distinct risk groups: low, intermediate, and high. A stepwise regression was employed, guided by AIC minimization of Cox proportional hazards models. The starting model included all continuous variables listed in **Supplemetary Fig. 1** and binary clinical features: sex, presence of massive mediastinal tumor (MMT), extranodal involvement, high erythrocyte sedimentation rate (ESR), involvement of more than three nodal regions, and symptomatic status (A/B). Only complete cases (N = 157) were used for the stepwise regression due to its algorithm.

The optimal model, yielding the lowest AIC, comprised the following variables: Age, Lymphocyte percentage, Albumin level, and Extranodal involvement. Lymphocyte percentage was excluded from the final model due to its violation of the proportional hazards assumption and lack of statistical significance. Although its removal resulted in a slightly increased Akaike Information Criterion (AIC), it ensured the model’s validity. This refined version is referred to as **Model 1**.

Although cytokine markers (TARC, sCD30, sCD163, sIL-6) were not selected through stepwise regression, they were retained for further analysis based on their established biological relevance. Cytokines are known to reflect immune activation and features of the tumor microenvironment in cHL, making them potentially important prognostic indicators, even if not initially selected by automated statistical procedures.

Given that raw cytokine levels are continuous variables, we sought to dichotomize them (low/high) to optimize PFS group differentiation. Cutpoints were determined by maximizing the log-rank statistic while ensuring a minimum group size to preserve clinical relevance. The minimum group size was controlled by the ‘minprop’ parameter, which defines the smallest allowable proportion of observations in each group (ranging from 0 to 0.5). While smaller values of ‘minprop’ can enhance group separation in Kaplan-Meier analysis, they may result in very small subgroups, limiting interpretability and practical use in subsequent analyses.

To minimize subjectivity in the choice of ‘minprop’, we performed a sensitivity analysis exploring a range of values. **Supplemetary Fig.5-8** illustrate this process for each cytokine, showing how the optimal cutpoint and corresponding p-value vary with ‘minprop’.

**Supplemetary Fig.5 Minprop sensitivity analysis for TARC**

**.
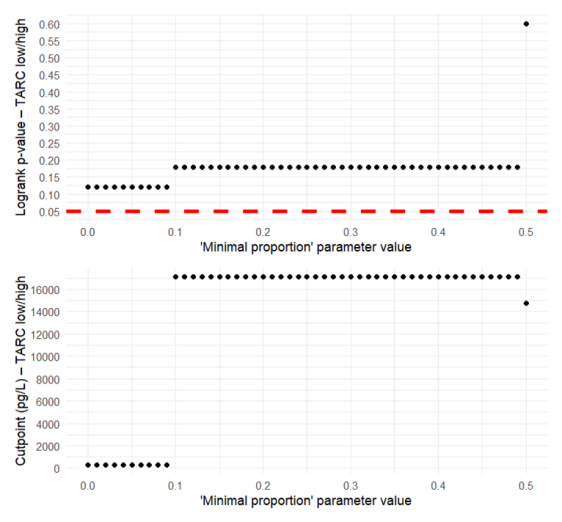
**

**Supplemetary Fig.6 Minprop sensitivity analysis for sCD30.**

**
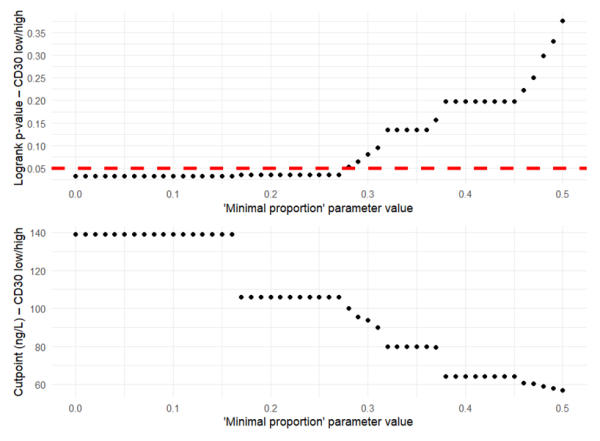
**

**Supplemetary Fig.7 Minprop sensitivity analysis for CD163.**

**
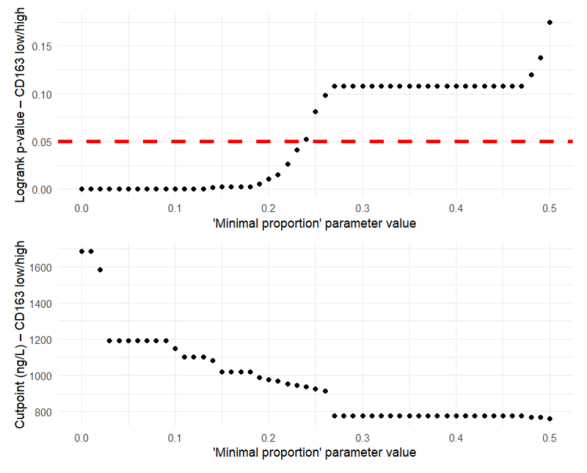
**

**Supplemetary Fig.8 Minprop sensitivity analysis for IL-6.**

**
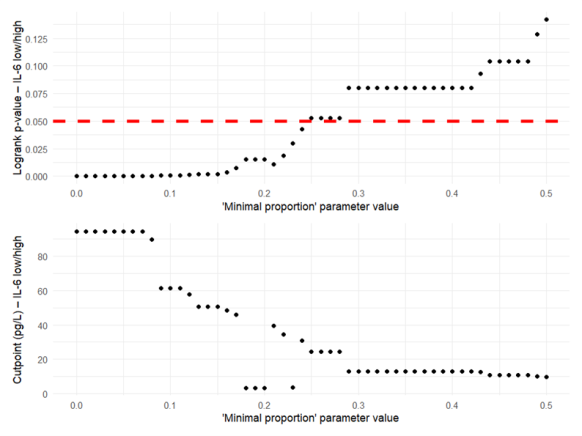
**

TARC was excluded from further consideration due to its failure to achieve statistically significant separation under any cutpoint strategy. For the remaining cytokines, the final thresholds were determined using two approaches: (i) selecting the maximum minprop yielding a p-value below the Bonferroni-corrected significance level (0.05/3), or (ii) selecting the maximum minprop yielding a minimal p-value if no threshold passed the corrected level but remained significant at the 0.05 level. The final cytokine cutpoints are shown in **Supplemetary Table 2**.

**Supplementary Table 2. Cytokine cutpoints.**

| **Cytokine** | **Cutpoint** |
| --- | --- |
| sCD30 (ng/L) | 139.1 |
| sCD163 (ng/L) | 970.5 |
| sIL-6 (pg/L) | 39.4 |

Despite including these categorized cytokines into a new stepwise regression, the resulting model closely resembled the previous iteration, retaining age, extranodal involvement, and albumin level. Although leukocyte count entered the model in place of lymphocyte percentage during this run, it was ultimately excluded due to lack of statistical significance, mirroring the earlier exclusion of lymphocyte levels. Survival analysis revealed that patients with elevated levels of both sCD163 and sIL-6 or sCD30 and sIL-6 experienced significantly worse PFS than other combinations **(Supplemetary Fig.9).**

**Supplemetary Fig.9 Progression-free survival for cytokine combinations**. (A) Progression-free survival for combination of sCD163 and sIL-6. (B) Progression-free survival for combination of sCD30 and sIL-6.


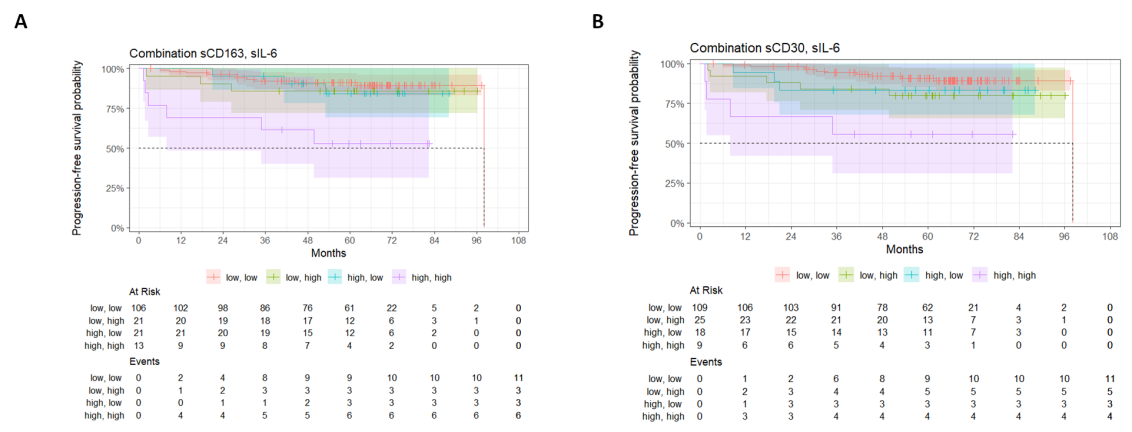


Although these “high–high” cytokine combinations were not significant when added to **Model 1**, their prognostic signal may be masked by collinearity with albumin levels. To further explore this, two alternative models were developed by substituting albumin with cytokine combinations:

- **Model 2:** Age, extranodal involvement, sCD163/sIL-6 high–high combination
- **Model 3:** Age, extranodal involvement, sCD30/sIL-6 high–high combination

These models demonstrated comparable AIC and similar C-index values compared to Model 1 and retained strong discriminative capability. Forest plots for these models are presented in **Supplemetary Fig.10-11** These models were trained using the available complete dataset specific to the included covariates. As a result, the number of observations (N) may differ across models. A unified cohort was used for fair comparison in cross-validation.

**Supplemetary Fig.10 Forrest plot of the HRs for Model 2.**

**
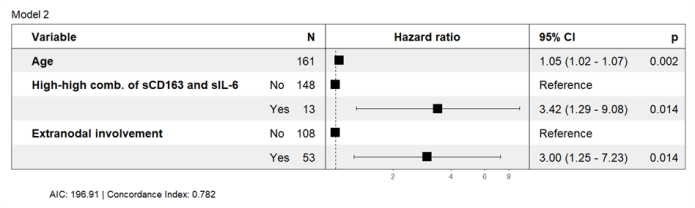
**

**Supplemetary Fig.11 Forrest plot of the HRs for Model 3.**

**
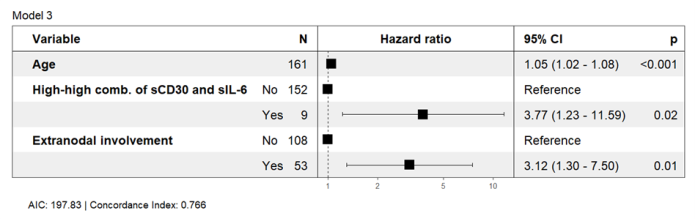
**

*4.1 Model cross-validation*

We evaluated the following Cox models using 10-fold cross-validation with the pec R package:

- **Model 1:** Age, extranodal involvement, albumin
- **Model 2:** Age, extranodal involvement, sCD163/sIL-6 high–high combination
- **Model 3:** Age, extranodal involvement, sCD30/sIL-6 high–high combination
- **Model 4 (Benchmark):** IPS-3 score only

Cross-validation was conducted on 159 patients with complete data across variables used in Models 1 to 4. Evaluation metrics included Integrated Brier Score (IBS) and Harrell’s C-index, both calculated on training data and through cross-validation. Methodological details align with Graf et al. [1] and Mogensen et al. [2].

Results are present in **Supplemetary Fig.12** with cross-validated prediction error curves, **Table 1.** with IBS and C-index, and **Supplemetary Fig.13.**

**Supplemetary Fig.12** **Cross-validated prediction error curves of the tested Models.**

**
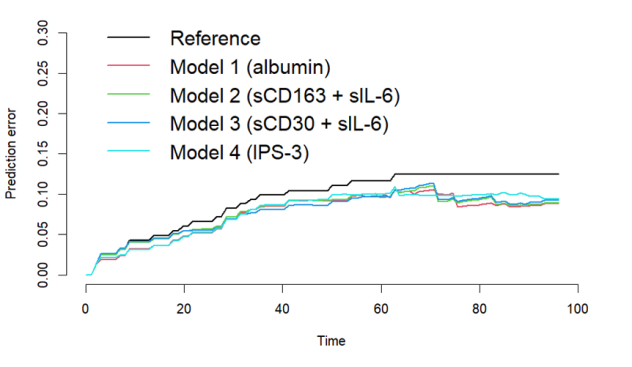
**

Based on the cross-validation results, the differences in predictive performance between the models were modest. However, these findings suggest that it may be appropriate to consider replacing the IPS-3 score with one of the newly proposed models (Model 1–Model 3), which offer comparable or improved prognostic utility.

**Supplemetary Fig.13 Comparison of diagnostic accuracy and positive and negative predictive values in predicting PFS events within 5 years.**


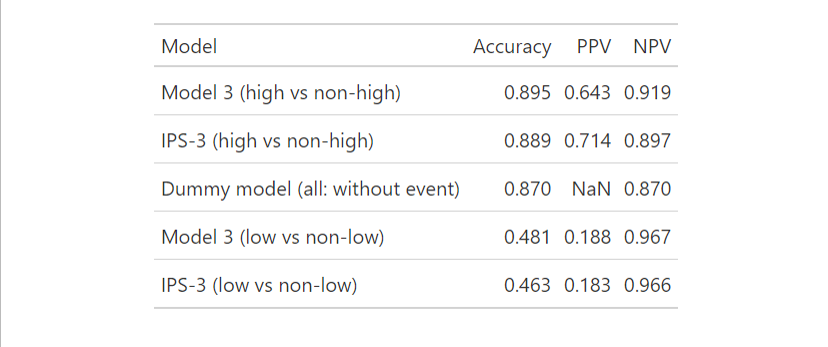


Abbreviations: PPV = positive predictive value; NPV = negative predictive value.

*4.2. Risk Stratification into Three Risk Groups*

To facilitate clinical application, linear predictors from Models 1–3 (which were trained again on a full dataset after CV) were discretized into three risk categories using the rsolr12() function from the rolr R package, implementing the SOL-1 and SOL-2 algorithms as described by Crowley et al.[3]. A minimum subgroup size of 10% (n = 16) was targeted to ensure robustness.

Among all discretization results, Model 3 using the SOL-2 algorithm demonstrated the best separation in Kaplan–Meier survival estimates and adjusted pairwise logrank tests (adapted for multiple comparison). Interestingly, despite being the best-performing continuous model, Model 1 failed to produce significantly distinct survival curves when discretized.

**Supplemetary references:**

1. Graf E, Schmoor C, Sauerbrei W, Schumacher M. Assessment and comparison of prognostic classification schemes for survival data. Stat Med. 1999 Sep 15-30;18(17-18):2529-45. doi: 10.1002/(sici)1097-0258(19990915/30)18:17/18<2529::aid-sim274>3.0.co;2-5. PMID: 10474158.
2. Mogensen UB, Ishwaran H, Gerds TA. Evaluating Random Forests for Survival Analysis using Prediction Error Curves. J Stat Softw. 2012 Sep;50(11):1-23. doi: 10.18637/jss.v050.i11. PMID: 25317082; PMCID: PMC4194196.
3. Qu P, Crowley J. rolr: Finding Optimal Three-Group Splits Based on a Survival Outcome. R package version 1.0.0, 2017. Available from: <https://cran.r-project.org/web/packages/rolr/rolr.pdf>
